# Applications of Exosomes in Female Medicine: A Systematic Review of Molecular Biology, Diagnostic and Therapeutic Perspectives

**DOI:** 10.3390/ijms27010504

**Published:** 2026-01-03

**Authors:** Heidi Mariadas, Jie-Hong Chen, Kuo-Hu Chen

**Affiliations:** 1 Department of Obstetrics and Gynecology, Taipei Tzu-Chi Hospital, The Buddhist Tzu-Chi Medical Foundation, New Taipei City 23142, Taiwan; heidimariadas@gmail.com; 2 School of Medicine, College of Medicine, MacKay Medical University, New Taipei City 25245, Taiwan; albertjhc@gmail.com; 3 School of Medicine, Tzu-Chi University, Hualien 97004, Taiwan

**Keywords:** exosomes, extracellular vesicles, biomarkers, obstetrics and gynecology, precision medicine

## Abstract

Exosomes are nanoscale extracellular vesicles that mediate intercellular communication by transporting microRNAs, proteins, and lipids. Generated through Endosomal Sorting Complex Required for Transport (ESCRT)-dependent mechanisms or ESCRT-independent pathways, exosomes are released when multivesicular bodies fuse with the plasma membrane. The ESCRT-dependent pathway involves sequential protein complexes (ESCRT-0, I, II, III) that recognize and sort ubiquitinated cargo, induce membrane budding, and facilitate vesicle scission. In contrast, the ESCRT-independent pathway relies on membrane lipids such as ceramide and proteins like tetraspanins (CD9, CD63, CD81) to promote vesicle formation without ESCRT machinery. Furthermore, post-translational modifications, including ubiquitination, sumoylation, and phosphorylation, further serve as molecular switches, modulating the affinity of ESCRT complexes or cargo proteins for membrane domains and affecting ILV formation rates. In reproductive medicine, exosomes regulate oocyte maturation, embryo–endometrial crosstalk, placental development, and maternal–fetal communication. Altered exosomal signaling contributes to obstetric complications, including preeclampsia, gestational diabetes mellitus, and preterm birth, whereas distinct exosomal miRNA signatures serve as potential diagnostic biomarkers. In gynecology, dysregulated exosomes are implicated in endometriosis, polycystic ovary syndrome, premature ovarian insufficiency, and gynecological malignancies. In contrast, mesenchymal stem cell-derived exosomes show therapeutic promise in restoring ovarian function and enhancing fertility outcomes. The distinctive molecular profiles of circulating exosomes enable minimally invasive diagnosis, while their biocompatibility and ability to cross biological barriers position them as vehicles for targeted drug delivery. Characterization of accessible data provides non-invasive opportunities for disease monitoring. However, clinical translation faces challenges, including standardization of isolation protocols, establishment of reference ranges for biomarkers, and optimization of therapeutic dosing. This review summarizes exosome biogenesis, characterization methods, physiological functions, and clinical applications in obstetrics and gynecology, with an emphasis on their diagnostic and therapeutic potential. Future directions include large-scale biomarker validation studies, engineering approaches to enhance exosome targeting, and integration with precision medicine platforms to advance personalized reproductive healthcare.

## 1. Introduction

Within the realm of cell biology and translational science, exosomes have transitioned from obscure cellular byproducts to major constituents of intercellular signal transduction and systemic communication networks [1]. Since their initial description as vesicles of endosomal origin released during reticulocyte maturation, exosomes have been identified as versatile carriers of biologically active contents, including nucleic acids (e.g., micro RNAs, messenger RNAs, and DNAs), proteins (e.g., tetraspanins, heat shock proteins, MHC molecules), and lipids (cholesterol, sphingomyelin, ceramides, and phosphatidylserine) [2,3]. Their highly dynamic and context-dependent roles extend beyond simple cargo transfer; exosomes are implicated in modulating immune responses, facilitating tissue regeneration, remodeling the extracellular matrix, and even promoting pathological processes such as tumor progression and metastasis [4,5,6]. Increasing evidence indicates that exosome-mediated signaling can be tightly regulated by both the cell of origin and environmental factors, including hypoxia, inflammation, and oxidative stress.

Advances in sequencing, imaging, and nanotechnology have rapidly expanded our understanding of exosome diversity and molecular function [7,8]. This surge of insight is matched by a growing number of clinical trials testing exosome biomarkers for early disease detection—such as in liver disease, breast cancer, gastrointestinal cancer, glioma, and prostate cancer, among others [9,10,11,12,13,14,15,16,17]—and by their translation into novel diagnostic and therapeutic strategies across multiple conditions.

This review summarizes recent advances in exosome biogenesis, molecular characterization methods, physiological functions, and—most importantly—their clinical relevance in obstetrics and gynecology (OB/GYN) [18,19,20,21]. Special emphasis is placed on how exosomes reshape diagnostic strategies, therapeutic approaches, and aging in reproductive medicine and women’s health [22,23,24,25]. By addressing their emerging roles in infertility, implantation disorders, obstetric complications, and gynecologic malignancies, this review underscores the central importance of exosome-mediated cell–cell crosstalk and the development of new, targeted biomarkers and treatments specific to OBGYN conditions, thereby opening new frontiers for precision reproductive care [26,27,28,29,30,31].

## 2. Method

**Figure 1:**
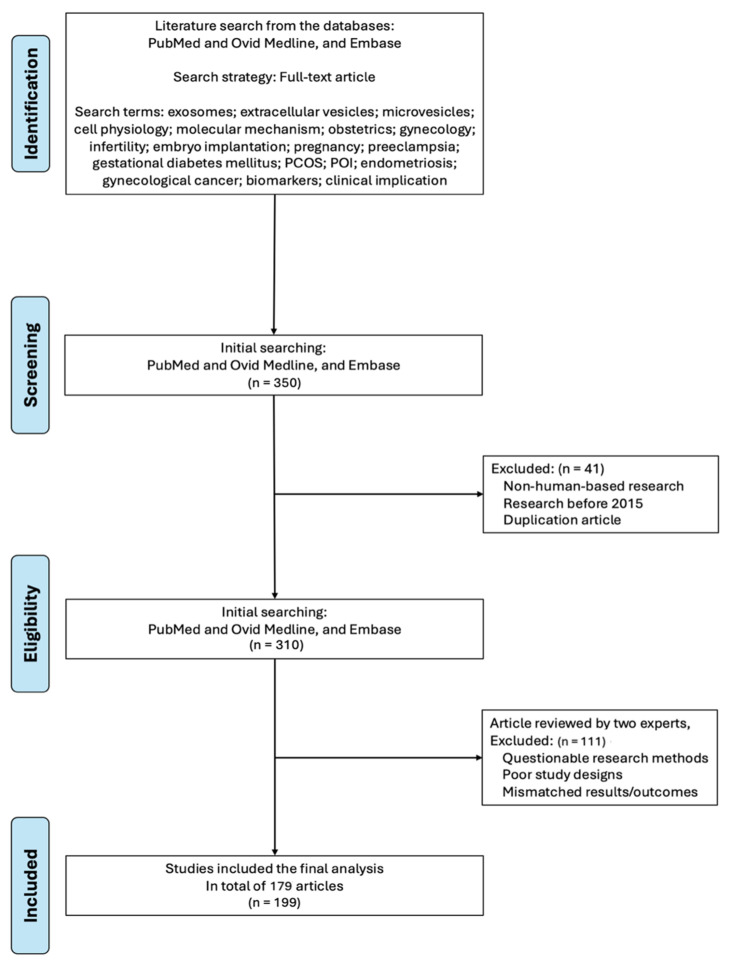
A flowchart of the database search, screening, selection, and inclusion of eligible articles from the literature.

This comprehensive review was conducted following the Preferred Reporting Items for Systematic Reviews and Meta-Analyses (PRISMA) guidelines to synthesize current knowledge on exosome molecular biology, cell physiology, and clinical applications in obstetrics and gynecology. The systematic review was registered in the public registration site OSF (registration number: 10.17605/OSF.IO/PVR8M at the website: https://archive.org/details/osf-registrations-pvr8m-v1, accessed on 19 November 2025). A systematic literature search was performed across multiple databases, including PubMed, Ovid Medline, and Embase, up to October 2025, prioritizing recent publications from the past five years while including seminal earlier studies for foundational concepts. A flowchart of the literature review and paper retrieval is shown in Figure 1.

The search strategy employed combinations of Medical Subject Headings (MeSH) terms and keywords including: “exosomes”; “extracellular vesicles”; “microvesicles”; “cell physiology”; “molecular mechanism”; “obstetrics; gynecology”; “infertility”; “embryo implantation”; “pregnancy”; “preeclampsia”; “gestational diabetes mellitus”; “polycystic ovary disease (PCOS)”; “primary ovarian insufficiency (POI)”; “endometriosis”; “gynecological cancer”; “biomarkers”; and “clinical implication”. Boolean operators (AND, OR) were used to refine searches and capture relevant literature. The inclusion criteria are (1) peer-reviewed original research articles, systematic reviews, and meta-analyses; (2) studies investigating exosome biogenesis, characterization, or function; (3) research focused on reproductive tissues, pregnancy, or gynecological conditions; (4) human studies; (5) publications in English. The exclusion criteria include: (1) conference abstracts without full-text availability; (2) animal model studies; (3) case reports with fewer than five patients; (4) studies lacking adequate methodological detail; (5) duplicate publications.

In the next stage, two independent reviewers inspected titles and abstracts of collected papers, followed by full-text evaluation of potentially relevant articles. Data extraction focused on exosome isolation methods, characterization techniques, molecular cargo profiles, physiological functions, diagnostic biomarker potential, therapeutic applications, and clinical trial outcomes. Quality assessment was performed using the Newcastle–Ottawa Scale for observational studies and the Cochrane Risk of Bias tool for interventional studies. To ensure the quality of the selected papers, there were two experts in the field responsible for reviewing papers, with one basic researcher (not one of the authors) and one clinician (one of the authors). Both experts had experiences of serving as reviewers for more than 50 journals, and they inspected these selected papers to review the research methods, study design and results or outcomes. Examples of “poor study design” or “questionable methods” included (but were not confine to) “no control group”, “selection bias in research participants”, “inappropriate selection of statistical methods”, etc. Examples of “mismatched results/outcomes” included “mismatch between the data and the theory”, “articles enrolled according to search terms (exosomes) did not report the results of the main research focus (exosomes)”, or “overstatements of research results”. A total of 350 articles were identified in the beginning, with 199 studies meeting the inclusion criteria after screening and quality assessment focused on human basic and clinical studies (Figure 1).

## 3. Results and Discussion

### 3.1. Exosome Characteristic and Biogenesis

**Figure 2:**
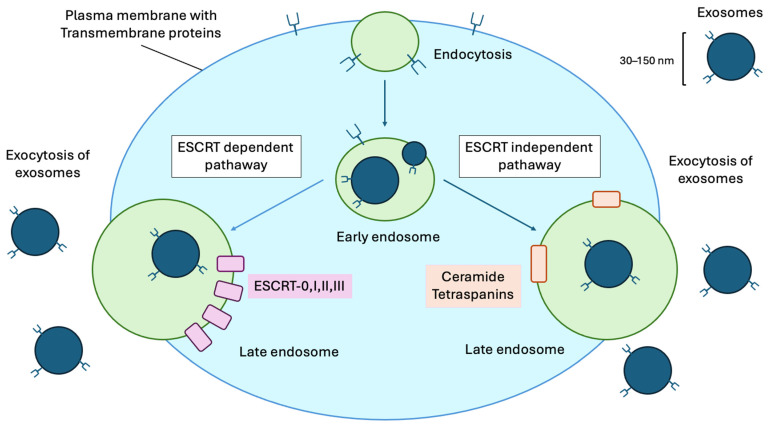
Exosome Biogenesis and Release Pathways. Exosomes (30–150 nm) are generated through endocytosis and maturation of early endosomes into late endosomes (green) via ESCRT-dependent (utilizing ESCRT-0, I, II, III complexes) or ESCRT-independent (Ceramide- and Tetraspanin-mediated) pathways, followed by fusion with the plasma membrane (blue) and exocytosis of intraluminal vesicles into the extracellular space.

Exosomes are small extracellular vesicles (30–150 nm) released by cells through a tightly regulated process that begins with endocytosis at the plasma membrane [1]. Internalized molecules and membrane components form early endosomes, which mature into late endosomes or multivesicular bodies (MVBs). Within MVBs, intraluminal vesicles are generated via two distinct Endosomal Sorting Complex Required for Transport (ESCRT) pathways: ESCRT-dependent and ESCRT-independent pathways [27,32] (Figure 2).

In addition to exosomes, cells release other vesicles, such as microvesicles (ectosomes), which bud directly from the plasma membrane. Distinct from the endosomal origin of exosomes. However, isolating pure exosome populations remains technically challenging due to overlapping physical properties. Consequently, the majority of current research analyzes mixtures of these subtypes, collectively referring to them as extracellular vesicles (EVs) [33,34,35].

The ESCRT-dependent pathway involves sequential protein complexes (ESCRT-0, I, II, III) that recognize and sort ubiquitinated cargo, induce membrane budding, and facilitate vesicle scission. In contrast, the ESCRT-independent pathway relies on membrane lipids such as ceramide and proteins like tetraspanins (CD9, CD63, CD81) to promote vesicle formation without ESCRT machinery. Understanding these pathways is fundamental to elucidating the biogenesis of exosomes in reproductive tissues, while altered exosome production can signal disease or physiological changes relevant to obstetrics and gynecology.

#### 3.1.1. ESCRT-Dependent Pathway

The ESCRT-dependent pathway is a highly orchestrated multi-step process that ensures precise cargo selection and vesicle formation within the endosomal system. It involves four main protein complexes—ESCRT-0, ESCRT-I, ESCRT-II, and ESCRT-III—alongside several auxiliary molecules and accessory proteins. ESCRT-0 initiates the process by recognizing and clustering mono- and polyubiquitinated membrane proteins, which serve as molecular tags for sorting. This recognition is primarily mediated by subunits such as HRS and STAM in ESCRT-0, which bind to both ubiquitin and phosphatidylinositol 3-phosphate on endosomal membranes [22,36]. The recruitment of ESCRT-I (containing proteins such as TSG101 and VPS28) links initial cargo clustering to vesicle budding; these proteins serve as adaptors, further concentrating ubiquitin-tagged cargos at specific endosomal microdomains [37,38]. Subsequently, ESCRT-II (with core components EAP20, EAP30, and VPS36) is recruited to facilitate membrane invagination, further sculpting the endosomal membrane to promote budding into the endosome’s lumen [39,40]. This inward budding generates intraluminal vesicles (ILVs) that become exosomes upon release. ESCRT-III, composed of CHMP family proteins, finalizes the vesicle scission through the assembly of spiral filaments, which constrict and cleave the membrane neck. The disassembly and recycling of ESCRT-III are driven by the AAA-ATPase VPS4, ensuring the repeated use of the complex [41]. Accessory molecules, such as ALIX, Bro1-domain proteins, and syntenin, also contribute to cargo recognition, membrane shaping, and regulation of ESCRT activity and exhibit tissue-specific roles in physiological processes, including placental development and oocyte maturation [8,42,43]. In reproductive tissues, the function of ESCRT proteins has emerged as a crucial determinant of exosome molecular composition. Experimental evidence indicates that dysregulation or mutations in ESCRT components can alter exosome secretion, size, and cargo diversity in the placenta, endometrium, and ovarian somatic cells. For instance, aberrant expression of ALIX or TSG101 may impair exosome release in trophoblast cells, thereby affecting trophoblast–endothelial interactions responsible for proper placental angiogenesis—and thereby contributing to conditions such as preeclampsia or fetal growth restriction [8,37,38,42,43].

#### 3.1.2. ESCRT-Independent Pathway

In parallel, the ESCRT-independent pathway constitutes a complementary mechanism that does not require the canonical ESCRT machinery but instead capitalizes on changes in membrane lipids and tetraspanin-enriched microdomains. The formation of ceramide from sphingomyelin, catalyzed by neutral sphingomyelinase 2 (nSMase2), is a key event. Ceramide induces negative membrane curvature by promoting microdomain formation, inward budding of the endosomal membrane, and ILV generation. This lipid-driven mechanism is particularly significant in reproductive cells with high metabolic and signaling demands, such as growing oocytes, granulosa cells, and invading cytotrophoblasts. Tetraspanins—including CD9, CD63, and CD81—act as molecular organizers within the membrane, clustering together specific entry and cargo proteins, and modulating the local lipid environment to promote vesiculation [3,43,44,45,46,47,48]. These tetraspanin microdomains facilitate the sorting of select proteins and RNAs into exosomes, contributing to cell-type-specific “exosomal signatures” relevant to OBGYN. For example, exosomes derived from endometrial stromal cells are enriched for CD9 and CD81, which correlates with their ability to modulate embryo implantation and endometrial receptivity [45].

Characterization of exosomes employs integrated techniques. Nanoparticle tracking analysis (NTA) and tunable resistive pulse sensing (TRPS) measure vesicle size and concentration, while transmission electron microscopy provides morphological confirmation. Protein markers such as tetraspanins (CD9, CD63) and cytosolic proteins (TSG101, Alix) are used to validate exosome identity. Molecular profiling through proteomics, transcriptomics, and lipidomics reveals cargo content significant for diagnostic and therapeutic applications.

#### 3.1.3. Accessory and Post-Translational Regulation

Recent molecular studies incorporating omics approaches—proteomics, transcriptomics, and lipidomics—demonstrate that exosome content is not static but dynamically regulated by cell-type, cell-state, and external cues such as hypoxia, hormonal changes, and inflammatory signals [4,38,49,50,51,52,53,54,55,56,57,58,59,60]. These influences can reshape the landscape of exosomal RNAs (miRNAs, lncRNAs, circRNAs), DNA fragments, proteins, and metabolites, collectively dictating their capacity to influence physiological functions or pathological processes [5]. In pregnancy, for example, alterations in exosome biogenesis in response to hypoxic stress may facilitate maternal adaptation but also contribute to the pathogenesis of gestational diseases [61,62,63,64].

Post-translational modifications, including ubiquitination, sumoylation, and phosphorylation, further serve as molecular switches, modulating the affinity of ESCRT complexes or cargo proteins for membrane domains and affecting ILV formation rates [3,65,66,67,68]. Lysosomal acidification and the balance between degradation and secretion pathways can also regulate MVB fate, thus influencing the number and composition of released exosomes under physiological versus pathological contexts, such as endometriosis, PCOS, or trophoblast invasion disorders [30,62].

The heterogeneity of exosomes is further magnified by the diversity of their cellular origins: immune cell exosomes display antigenic peptides or MHC complexes, whereas cancer-derived exosomes frequently package protumoral miRNAs and proteins [6,69]. Recent advances have enabled the detection and characterization of exosomes in obstetric and gynecologic clinical samples, including maternal blood, placental tissue, amniotic fluid, and follicular fluid. These insights enhance our ability to utilize exosomes as biomarkers and therapeutic vehicles in reproductive medicine, offering novel avenues for the diagnosis and treatment of pregnancy complications and gynecological disorders. This selective enrichment forms the foundation for the diagnostic application of exosomal “signatures”—the molecular fingerprints that foreshadow or reflect disease processes [70,71]. The complexity and adaptability of these molecular mechanisms, including cellular source and state, underscore the elaborate network that controls exosome biogenesis, sorting, and release, ultimately determining their biological activity in health and disease.

### 3.2. Cell Physiology and Intercellular Communication

#### 3.2.1. Physiological Functions

Exosomes are critical mediators of physiological processes essential for reproductive health. Beyond cell-to-cell signaling, exosomes are now recognized as fine-tuners of fundamental cellular physiology in the reproductive system. They facilitate intercellular communication within the female reproductive tract, coordinating cellular responses for homeostasis, immune regulation, tissue remodeling, and supporting reproductive functions in both obstetrics and gynecology [72,73,74,75,76]. In pregnancy, exosomes contribute to immune modulation at the maternal–fetal interface, supporting tolerance of the semi-allogenic fetus and mediating vascular remodeling necessary for placental development.

Mechanistically, exosomes deliver functional cargo—proteins, RNAs, lipids—to recipient cells via membrane fusion, endocytosis, or receptor-mediated uptake [72,73,74,75,76]. This horizontal transfer alters gene expression and signal transduction in target cells. In OB/GYN contexts, such communication regulates follicle maturation, embryo implantation, placentation, and labor processes.

Additionally, exosomes play roles in metabolic crosstalk, stem cell niche maintenance, and modulation of apoptosis within reproductive tissues. Their systemic influence is evident in pregnancy maintenance and adaptations, underscoring their potential as biomarkers of physiological status and therapeutic targets.

#### 3.2.2. Roles in Obstetrics

Within the female reproductive tract during pregnancy, exosomes facilitate complex intercellular communication that coordinates tissue remodeling, immune regulation, and vascular adaptation, all of which are essential for fetal development [72,73,74]. Endometrial exosomes mediate embryo–endometrium crosstalk that determines implantation success [77,78]. Their molecular cargos—adhesion molecules, matrix metalloproteinases, and regulatory RNAs—enhance trophoblast adhesion and invasion, modulate the local immune milieu, and facilitate extracellular matrix remodeling [77,79]. These exosomal interactions are vital for establishing uterine receptivity and maintaining early pregnancy.

During embryo implantation, exosomes derived from endometrial epithelial cells and trophoblasts carry molecular signals that facilitate crosstalk between the maternal endometrium and the developing embryo. These vesicles enrich the local milieu with proteins such as fibulin1 (FBLN1), cysteine-rich 61 (CYR61), and complement regulators, all of which contribute to tissue remodeling and successful trophoblast invasion. They regulate endothelial cell proliferation, migration, and tube formation by delivering pro-angiogenic microRNAs such as miR-210, miR-126m and functionally modulate key signaling pathways, such as JAK-STAT, MAPK, and vascular endothelial growth factor (VEGF), critical for cell adhesion, angiogenesis, and the creation of an immunotolerant environment within the uterus [29,53,80,81,82,83,84]. During the peri-implantation period, specific exosomal microRNAs (e.g., bta-miR-98, hsa-miR-30d) regulate immune gene expression and facilitate endometrial receptivity and embryo adhesion, thereby preventing rejection while maintaining immune surveillance [77].

Exosomes found in maternal serum and amniotic fluid reflect dynamic changes in pregnancy physiology, and their molecular profiles correlate with pregnancy outcomes [59,63,75,85,86,87]. Dysregulation of exosomal content is linked to obstetric complications, including preeclampsia and gestational diabetes mellitus (GDM), in which altered exosomal microRNA signatures mediate inflammatory responses, insulin resistance, and endothelial dysfunction [37,53,88]. Exosomes also contribute to labor by regulating inflammatory pathways and uterine contractions. Their cargos can stimulate cytokine production and remodeling of the cervical extracellular matrix, thereby facilitating parturition timing and contributing to normal and preterm labor processes [59,89,90].

#### 3.2.3. Roles in Gynecology and Reproductive Endocrinology

In ovarian follicles, exosome-mediated transfer of microRNAs and proteins regulates granulosa cell functions, influencing steroidogenesis, cellular proliferation, oocyte maturation, ovarian function, and apoptosis [91,92,93,94,95]. For example, exosomal miR-224 has been shown to stimulate estradiol secretion by increasing CYP19A1 expression, thereby directly promoting follicular growth and oocyte maturation, which are crucial for female fertility [96]. Other exosomal miRNAs and regulatory factors modulate granulosa cell proliferation and survival, demonstrating both autocrine and paracrine effects within the ovarian microenvironment [94,97].

Exosomes influence metabolic and inflammatory states in gynecological pathologies. In polycystic ovary syndrome (PCOS), altered exosome secretion and cargo composition contribute to granulosa cell dysfunction and ovarian inflammation [39,91,92,98,99]. In endometriosis, exosomes derived from ectopic endometrial tissue modify immune cells and fibroblasts in the pelvic environment, thereby supporting lesion survival and fibrosis [32,100,101,102,103].

Moreover, exosomes influence the maintenance of tissue stem cell niches and can promote tissue regeneration. In ovarian and endometrial tissues, exosomal signals can stimulate proliferation and differentiation, thereby aiding post-injury repair or cyclical uterine remodeling [65,104,105,106]. Apoptosis regulation is also governed by exosome content; for example, exosomal proteins in uterine fluid across different phases of the cycle may tip the balance between cell survival and programmed cell death, thereby ensuring timely shedding and regeneration of the endometrium.

Emerging evidence also highlights the role of exosomes in gynecologic malignancies. Tumor-derived exosomes mediate immune evasion, promote metastasis, and confer chemoresistance through the transfer of oncogenic microRNAs and proteins [31,44,107,108,109,110,111]. The unique molecular signatures of tumor exosomes provide promising diagnostic biomarkers and therapeutic targets [44,112].

#### 3.2.4. Intercellular Communication

Exosome-mediated intercellular communication represents a transformative paradigm for understanding how cells coordinate complex biological activities across tissues and organs [94,97]. Unlike traditional soluble factors, exosomes deliver their biologically active cargoes with high efficiency and specificity and are protected by their lipid bilayer membranes. Recipient cells internalize exosomes via multiple mechanisms, including membrane fusion, phagocytosis, micropinocytosis, and receptor-mediated endocytosis, thereby enabling the targeted delivery of proteins, RNAs, and lipids directly into the cytoplasm or specific cellular compartments [113,114,115].

This horizontal transfer of molecular information enables exosomes to modulate gene expression, reshape signaling pathways, and reprogram recipient cell behavior [116]. In oncology, tumor-derived exosomes transfer oncogenic microRNAs, proteins, and metabolites that promote tumor progression, immune evasion, metastasis, and therapeutic resistance [76,117]. Conversely, immune cell-derived exosomes can fine-tune immune responses, promoting either activation or tolerance, as physiological conditions dictate [118].

Exosomal transport of misfolded or pathogenic proteins also contributes to the spread of neurodegenerative diseases such as Alzheimer’s and Parkinson’s, underscoring their role in pathological intercellular networks [119]. Increasing evidence positions exosomes as both local and systemic mediators that profoundly influence tissue microenvironments, immune landscapes, and overall organismal health. As technologies evolve, detailed dissection of exosomal communication will illuminate their roles in physiology and disease, offering promising avenues for therapeutic intervention.

### 3.3. Clinical Applications in Obstetrics and Gynecology

**Figure 3:**
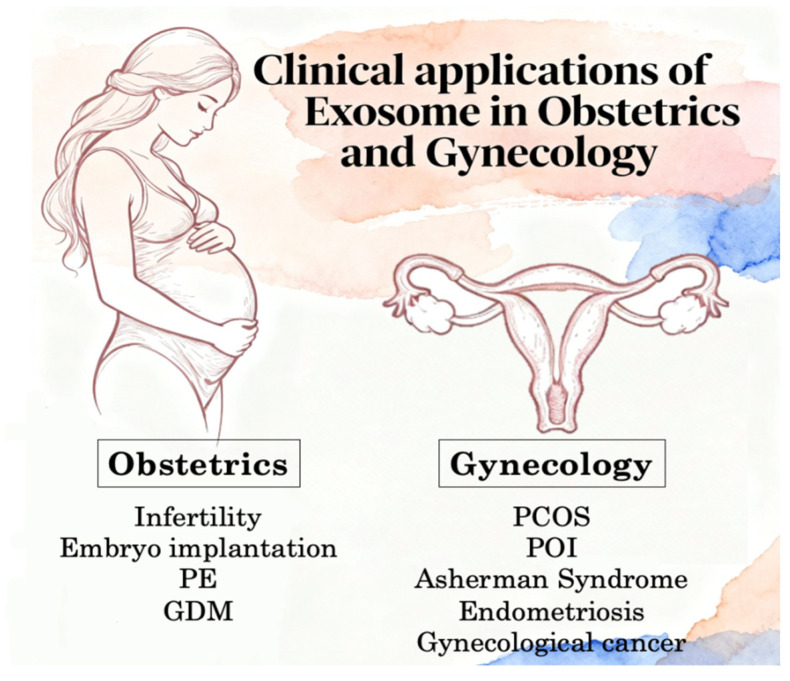
Exosomes play a pivotal role in the diagnosis and clinical management of obstetric and gynecological diseases. Gynecological cancers include ovarian cancer, uterine and endometrial cancer, and cervical cancer. GDM: Gestational diabetes mellitus; PCOS: Polycystic Ovary Syndrome; PE: Pre-eclampsia; POI: Premature Ovarian Insufficiency.

Exosomes play a pivotal role in the diagnosis and clinical management of obstetric and gynecological diseases. Gynecological cancers include ovarian cancer, uterine and endometrial cancer, and cervical cancer (Figure 3).

#### 3.3.1. Exosomes: Cellular Origin in Pregnancy, Standardization of Techniques, Diagnosis and Quantification

##### Cellular Origin in Normal Pregnancy

In normal pregnancy, the pool of circulating maternal extracellular vesicles (EVs) is a complex mixture derived from both maternal and fetal tissues. The syncytiotrophoblast (STB), which is in direct contact with maternal blood, is the primary source of placenta-derived exosomes (STB-EVs), releasing them into the maternal circulation as early as 6 weeks’ gestation to modulate maternal immune tolerance and vascular adaptation [120,121]. Other key contributors include maternal platelets, leukocytes, and endothelial cells, which collectively maintain systemic homeostasis. In pathological states, this cellular profile shifts dramatically. For instance, in preeclampsia (PE), placental oxidative stress and hypoxia trigger an exaggerated release of STB-EVs enriched with anti-angiogenic factors (e.g., sFlt-1) and inflammatory cytokines, alongside a surge in activated platelet- and endothelial-derived EVs that reflect systemic maternal endothelial dysfunction [122,123]. Similarly, in gestational diabetes mellitus (GDM), hyperglycemia stimulates the release of placental exosomes that carry distinct miRNAs (e.g., miR-320b), which impair insulin signaling and glucose tolerance [124].

##### Standardization of Techniques

Currently, there is no single “gold standard” clinical assay for evaluating exosomes in pregnancy, but the International Society for Extracellular Vesicles (ISEV) guidelines have established a rigorous research framework. The standard workflow now combines size-based isolation (e.g., ultracentrifugation, size-exclusion chromatography) with comprehensive characterization. This includes physical analysis via Nanoparticle Tracking Analysis (NTA) or Tunable Resistive Pulse Sensing (TRPS) to determine particle concentration and size, coupled with immunophenotyping (Western blot, flow cytometry) for classical EV markers (CD63, CD81, CD9) and placenta-specific markers such as placental alkaline phosphatase (PLAP) or syncytin-1 [122,125]. Recent high-impact studies emphasize that combining these methods is critical for distinguishing placenta-derived EVs from the vast background of maternal host EVs [121,125].

##### Diagnostic Value of Assays

Emerging literature allows for the ranking of EV analytical techniques based on their diagnostic utility for obstetric complications:

1. Omics-Based Profiling (Highest Value): High-throughput sequencing of exosomal cargo (miRNA, proteomic, lipidomic) offers the highest diagnostic specificity. Multi-marker panels from placenta-enriched EV fractions have demonstrated superior sensitivity for predicting PE and GDM compared to single-marker assays [121,124,125].
2. Cell-Specific Immunophenotyping: Assays targeting STB-specific surface markers (e.g., PLAP+ flow cytometry or ELISA) provide moderate-to-high diagnostic value by specifically quantifying the “fetal signal” amid maternal noise and correlate strongly with placental stress [122].
3. Functional Assays: In vitro assays that measure the bioactivity of isolated EVs on target cells (e.g., endothelial tube formation, monocyte activation) provide mechanistic insights but are currently too labor-intensive for routine clinical diagnostics [120,121].
4. Bulk Concentration and Size (Lowest Specificity): While total EV concentration often increases in pathology, it lacks specificity due to high inter-individual variability and the influence of non-pregnancy factors (e.g., BMI, inflammation), limiting its standalone diagnostic utility [125].

##### Significance of Exosome Quantification

Despite lower specificity than cargo analysis, the quantity of circulating exosomes holds significant diagnostic potential when stratified by gestational age. Longitudinal studies indicate that total EV and STB-EV concentrations increase progressively across normal gestation. A deviation from this trajectory—specifically, a sharp, early rise in STB-EVs—has been repeatedly identified as a hallmark of early-onset preeclampsia and typically precedes clinical symptoms [121,122,125]. Conversely, some studies in GDM have reported altered ratios of placental-to-total EVs, suggesting that the quantitative load reflects the magnitude of placental stress [122,124]. Thus, while “counting” exosomes alone is insufficient, quantitative changes serve as a powerful “red flag” that warrants deeper molecular investigation.

#### 3.3.2. Exosomes in Obstetrics Disease

##### Infertility

According to data from the World Health Organization (WHO), infertility affects approximately 17.5% of the adult population worldwide. Studies show that female factors alone account for at least 35% of all infertility cases, which is a multifactorial condition affecting about 10% of women of reproductive age globally [126]. A recent analysis from the Global Burden of Disease Study 2021 found that more than 110 million women are affected by female infertility [127]. Female infertility can result from dysfunctions within the ovarian follicle microenvironment, where exosomes in follicular fluid (FF) act as crucial mediators of cell communication necessary for folliculogenesis and oocyte quality [51,97,128,129]. Proteomic analyses of FF-derived exosomes reveal distinct protein signatures that vary between young, fertile women and aging, infertile women. Proteins that are differentially expressed (DEPs) linked to B-cell activation, immune responses, and disrupted metabolic processes are notably increased in aging women, correlating with poor follicular development and infertility [51]. Specific exosomal proteins, such as ENO1, HSP90B1, fetuin-B, complement component C7, CD9, and APOC4, are involved in regulating follicle development, indicating their potential as biomarkers for infertility diagnosis and as targets for treatment [51,130].

Exosomes released by oviductal epithelial cells (oEVs) improve embryo development by transferring bioactive molecules that support cell growth and decrease apoptosis [131]. These oviductal extracellular vesicles exhibit species-specific features and contain distinct protein and lipid profiles that facilitate communication between embryo and oviduct. In addition to FF exosomes, maternal circulating exosomal microRNAs have become highly predictive biomarkers for early recurrent pregnancy loss (RPL). Recent studies identified 43 miRNAs with altered expression in patients with ongoing pregnancies versus those with RPL, with miR-185-5p notably increased in RPL patients [84]. Mechanistically, exosomal miR-185-5p from trophoblast cells lowers VEGF levels in decidual natural killer (dNK) cells, disrupting angiogenesis at the maternal–fetal interface and possibly contributing to RPL development.

Furthermore, stem cell-derived exosomes, especially from mesenchymal stem cells (MSCs), are emerging as innovative therapeutic options to restore ovarian function by inhibiting cell death, promoting blood vessel growth, and regulating key internal pathways [132]. These exosomes carry miRNAs that influence granulosa cell proliferation and survival, offering a promising cell-free regenerative therapy for infertility related to ovarian failure or aging [132].

##### Embryo Implantation

Successful embryo implantation depends on tightly regulated communication between the maternal endometrium and the embryo, mediated by extracellular vesicles, including exosomes [133]. Endometrial epithelial cell-derived exosomes carry molecular cargos—such as proteins and RNAs (e.g., miR-150-5p, miR-150-3p, miR-149-5p, and miR-146b-3p)—that promote trophoblast adhesion, invasion, and migration, which are essential for implantation and may also influence ectopic pregnancy risk [134,135]. The composition of endometrial exosomes varies throughout the menstrual cycle, with certain exosomal subtypes increasing during the window of implantation.

Studies isolating exosomes from primary human endometrial epithelial cells (pHEECs) show a heterogeneous population of vesicles marked by proteins such as HSP70, TSG101, CD9, and CD81 [38]. Functional analyses demonstrate that these exosomes support trophoblast functions, while proteomic profiling identifies key proteins involved in cell signaling and adhesion pathways. These findings suggest that exosomal cargos could serve as promising biomarkers for implantation success and as therapeutic agents to enhance implantation rates, especially in assisted reproductive techniques where failure is common [136].

Moreover, seminal and amniotic fluids are important sources of exosomes in reproductive health. Seminal fluid contains exosome-borne signaling molecules that modulate the immune environment of the female reproductive tract, preparing it for embryo implantation and optimal placentation. In contrast, amniotic fluid is rich in fetal-derived exosomes that reflect fetal development and health [42,45]. Analyzing these fluids offers valuable clinical insights. Placenta-derived exosomes (pEXOs) are detectable in maternal blood as early as 6 weeks of gestation, and their levels increase with gestational age; this rise correlates with better pregnancy outcomes and fewer inflammatory issues like preeclampsia [19]. Additionally, examining amniotic fluid exosomal content enables noninvasive monitoring of fetal development and early detection of potential pregnancy complications [19,137].

##### Preeclampsia (PE)

Preeclampsia represents a serious obstetric complication characterized by pregnant hypertension and multi-organ dysfunction after 20 weeks of gestation, including initial abnormal placentation, progressive systemic inflammation, and eventual endothelial dysfunction [138,139,140]. Placental-derived exosome (PdE) levels are elevated in PE and carry distinct miRNA and protein signatures that reflect placental dysfunction [33,141]. Early pregnancy exosomal profiles can differentiate women at risk of developing PE. Specific exosomal microRNAs (e.g., hsa-miR-675-5p, hsa-miR-3614-5p, miR-520a-5p) and proteins associated with angiogenesis, migration, invasion, and coagulation pathways serve as novel biomarkers for early diagnosis of PE and for predicting disease severity, distinguishing early- and late-onset forms of PE [63,139,142,143,144]. These exosomal microRNAs originate from placental trophoblasts under hypoxic conditions and endothelial dysfunction, and function as hallmarks of preeclampsia pathophysiology [145].

Therapeutically, low-dose aspirin (LDA), the current preventive treatment for high-risk PE pregnancies, is hypothesized to exert its effects partly by modulating placental exosome release and composition, impacting maternal systemic and placental vascular function [141,144]. The analysis of exosomal content thus offers a noninvasive window into the pathophysiology of PE and a route for monitoring therapeutic interventions, potentially enabling personalized management strategies for PE [146,147,148].

##### Gestational Diabetes Mellitus (GDM)

Gestational diabetes mellitus (GDM) affects between 5% and 25% of pregnancies worldwide, with the International Diabetes Federation estimating that hyperglycemia in pregnancy impacts approximately 23 million births annually [149,150]. The wide range in prevalence is attributed to differences in diagnostic criteria and study populations.

GDM involves hyperglycemia during pregnancy with adverse maternal and fetal outcomes. Proteomic analyses comparing total plasma exosomes and placental-derived exosomes in patients with GDM and controls identified differentially expressed proteins involved in immunity, complement activation, inflammation, and coagulation. Placental exosomal miRNAs from the miR-99 family regulate trophoblast autophagy by targeting proteins like myotubularin-related protein 3 (MTMR3), influencing insulin resistance in trophoblasts [79,151,152,153]. Reduced miR-99 family expression in placental exosomes correlates with impaired autophagy and increased insulin resistance, implicating these miRNAs as therapeutic targets for GDM.

Studies also show that circRNAs in umbilical cord blood exosomes are differentially expressed in GDM and may influence both maternal glucose regulation and fetal development; many interact with miRNAs linked to metabolic pathways. These changes highlight the potential of exosomal circRNAs as biomarkers for GDM diagnosis and as contributors to the pathophysiology of GDM and fetal growth [154]. Exosomal proteins and miRNAs have potential as early predictive biomarkers for GDM, enabling timely intervention to mitigate associated risks [36,153]. Monitoring exosomal content postpartum may also assist in managing long-term GDM complications for mother and child [155].

#### 3.3.3. Exosomes in Gynecological Disease

##### Polycystic Ovary Syndrome (PCOS)

PCOS is a prevalent endocrine disorder marked by ovarian follicular arrest and hyperandrogenemia [156]. Follicular fluid exosomes in PCOS exhibit dysregulated cargo, including altered microRNA and protein profiles, that impair granulosa cell function and oocyte development [96]. Exosomal microRNAs, including miR-379-5p, miR-143-3p, miR-155-5p, miR-323-3p, etc., particularly miR-379-5p, are dysregulated in PCOS. Increased androgen exposure promotes the exosomal release of miR-379-5p from granulosa cells, reducing intracellular levels and decreasing cell proliferation in a follicular-stage-specific manner [99,157,158,159,160]. This disruption in granulosa cell function contributes to ovarian inflammation and follicular growth arrest characteristic of PCOS [160,161]. Studies of intraovarian and intravenous administration of these exosomes have restored ovarian morphology, normalized hormone levels, improved metabolic parameters, and enhanced fertility, suggesting potential as a novel clinical treatment for PCOS [92,132].

##### Premature Ovarian Failure/Insufficiency (POF/POI)

POI leads to early loss of ovarian function, represents a major cause of infertility, hypoestrogenism, and associated health risks. MSC-derived exosomes exhibit regenerative capabilities by promoting granulosa cell proliferation, inhibiting apoptosis, and stimulating angiogenesis in ovarian tissue [87,132]. These effects are mediated by modulation of pathways including the Hippo, SMAD3-AKT, and mitochondrial function [39,68,162,163].

Clinical studies using human umbilical cord MSC-derived exosomes in POI models have shown restoration of follicle number, normalization of serum hormone profiles, and improved reproductive outcomes [164]. Hypoxic preconditioning of MSCs further enhances exosomal angiogenic potential by targeting the PTEN-PI3K-AKT-mTOR pathway via miR-205-5p [61,165,166]. This evidence supports the clinical translation of exosomal therapy as a promising, minimally invasive alternative to hormone replacement for POI patients [93,132].

##### Endometriosis

According to the WHO, Endometriosis affects around 10% of reproductive-age women, which involves ectopic growth of endometrial tissue, causing chronic pain and infertility [100]. Exosomes secreted from peritoneal macrophages and endometrial cells facilitate lesion progression by transferring oncogenic long noncoding RNAs (lncRNAs) like CHL1-AS1 and miRNAs (e.g., miR-134-5p, miR-197-5p, miR-22-3p, miR-320a, miR-494-3p, and miR-939-5p) that regulate cellular proliferation, migration, immune evasion, and fibrosis [20,167,168,169]. These exosomes modulate macrophage polarization and influence the pathological niche supporting ectopic tissue survival.

Circulating and peritoneal fluid exosomes carry disease-specific molecular signatures, which may serve as potential noninvasive biomarkers for early diagnosis and monitoring of recurrence [170]. Targeting exosomal communication pathways such as the miR-610/MDM2 axis offers novel therapeutic strategies for halting disease progression and alleviating infertility linked to endometriosis [39,68,167].

##### Asherman Syndrome

Asherman syndrome, characterized by intrauterine adhesion (IUAs) formation, causes menstrual dysfunction and infertility through progressive endometrial fibrosis and decreased receptivity [104]. Mesenchymal stem cell-derived exosomes represent a novel regenerative approach, delivering anti-inflammatory molecules and growth factors that reduce uterine fibrosis, suppress pro-inflammatory pathways, and promote endometrial regeneration [164]. These exosomes facilitate the restoration of normal uterine architecture and the normalization of endometrial thickness by enhancing angiogenesis and stimulating endometrial stem cell proliferation.

Treatment with MSC-derived exosomes offers significant advantages over traditional surgical adhesiolysis approaches by addressing the underlying endometrial damage and fibrotic remodeling rather than merely providing mechanical separation of adhesions. By simultaneously promoting neovascularization, reversing the fibrotic phenotype, and restoring endometrial receptivity, exosome-based therapy restores functional capacity in uterine tissues. It improves fertility outcomes, with reduced recurrence rates compared with conventional surgical interventions.

#### 3.3.4. Gynecological Malignancies

##### Ovarian Cancer

Epithelial ovarian cancer (EOC) utilizes exosomes extensively to promote tumor progression and metastasis within the peritoneal cavity. EOC-derived exosomes modify the tumor microenvironment by delivering oncogenic proteins, Circular RNA (circFoxp1, Foxo3) significantly upregulated in OC cell line, circular RNAs (circRNAs), miRNAs (e.g., miR-221-3p, miR-34a, miR-4732-5p, miR-99a-5p), and lncRNAs (e.g., MALAT1, SNHG17) to recipient cells, including endothelial cells and macrophages, promoting angiogenesis, immune modulation, epithelial–mesenchymal transition, and drug resistance [21,64,112,171,172,173,174,175,176,177,178,179,180,181]. Exosomal cargos, such as CD44 and CD47, facilitate cancer cell invasion by remodeling mesothelial barriers [71,182].

Serous ovarian cancer (SOC) is the most common epithelial ovarian cancer subtype with varied responses to platinum chemotherapy, affecting patient survival. Analysis reveals that exosome-associated genes play key roles in SOC carcinogenesis and drug resistance. MiRNAs (miR-1290) and hub genes such as GNAI1, NCAPH, MMP9, AURKA, and EZH2 are potential targets for improving carboplatin sensitivity and prognostic prediction, highlighting the significance of exosomes in SOC progression and therapy resistance [183,184].

Proteomic and transcriptomic profiling of exosomes can identify differentially expressed miRNAs (DE-miRNAs) or subtype-specific biomarkers (e.g., PLAU, LAMB1) for aggressive ovarian cancer [58,185]. Clinically, exosomal miRNAs in serum and ascitic fluid are being investigated as noninvasive diagnostic and prognostic biomarkers [50,186,187]. Therapeutically, engineered exosomes that deliver anticancer drugs, such as triptolide-loaded exosomes, exhibit enhanced tumor targeting and reduced systemic toxicity, underscoring exosomes’ dual potential as biomarkers and drug-delivery vehicles in ovarian cancer management [46,188].

##### Uterine and Endometrial Cancer

Cancer-associated fibroblast (CAF)-derived exosomes carrying lncRNAs such as NEAT1 contribute to the progression of endometrial cancer by modulating key signaling pathways like STAT3-YKL-40 [189]. These exosomes enhance tumor cell proliferation and metastasis, with elevated exosomal biomolecules correlating with clinical tumor stage and histological grade.

Analysis of microparticles in uterine blood shows increased levels of tissue factor (TF), endothelial (CD144), and monocytic (CD14) microparticles in patients with endometrial cancer compared with controls [190,191]. These findings suggest that monocyte–macrophage-derived exosomes/microparticles may serve as novel diagnostic or prognostic markers for endometrial carcinogenesis, supporting the clinical utility of liquid biopsy using exosomal platforms [10,192].

##### Cervical Cancer

Exosomal miRNAs (e.g., miR-1286) and lncRNAs (e.g., lncRNA DLX6-AS1) are implicated in cervical cancer pathogenesis by influencing cell proliferation, invasion, immune escape, lymphangiogenesis, and metastasis. miR-22 is frequently downregulated in various cancers, including cervical cancer, and is associated with a poor prognosis in cervical cancer [193,194,195,196,197,198]. Patterns of exosomal molecular cargo differ between cancerous and normal tissues, and these vesicles can be detected in body fluids, providing biomarkers for early diagnosis and disease monitoring [195].

Therapeutic approaches targeting exosomal communication or engineered exosomes for drug delivery are under investigation to disrupt cervical cancer progression and enhance treatment efficacy [44,197].

### 3.4. Exosomal Biomarkers in OB/GYN Diagnostics and Therapeutic Applications

The unique molecular signatures of circulating exosomes provide unprecedented opportunities for noninvasive diagnosis, prognostication, and disease monitoring in obstetrics and gynecology. Exosomes isolated from readily accessible biofluids, including blood plasma, serum, urine, follicular fluid, peritoneal fluid, and amniotic fluid, reflect the physiological and pathological states of reproductive organs and tissues (Table 1 and Table 2).

#### 3.4.1. Diagnostic Advantages

Exosomal biomarkers offer several distinct advantages over traditional diagnostic modalities: (1) noninvasive or minimally invasive sample collection from accessible biofluids; (2) exceptional stability of exosomal cargo protected by lipid bilayer membranes; (3) real-time reflection of tissue pathophysiology; (4) the ability to detect early disease before manifestation of clinical symptoms; (5) capability for monitoring treatment responses and disease progression; (6) tissue-specific exosome populations that can be selectively isolated and analyzed.

#### 3.4.2. Therapeutic Applications

Mesenchymal stem cell-derived exosomes represent a significant shift in regenerative medicine by offering a cell-free therapeutic method that bypasses the safety concerns and regulatory issues linked to live-cell therapies. Sourced from various tissues such as bone marrow, adipose tissue, umbilical cord, placenta, menstrual blood, and amniotic epithelium, these exosomes provide multiple therapeutic benefits through anti-apoptotic signaling via targeted microRNA delivery, strong immunomodulation and anti-inflammatory effects, stimulation of angiogenesis and tissue vascularization, support for parenchymal cell growth and differentiation, regulation of extracellular matrix remodeling, and restoration of mitochondrial function by reducing oxidative stress. Several clinical trials are underway to evaluate MSC-derived exosomes for treating premature ovarian insufficiency and other reproductive conditions, with early results showing excellent safety profiles and initial signs of effectiveness in restoring ovarian function, balancing hormonal levels, and improving fertility outcomes.

### 3.5. Discussion: Challenges and Future Directions

#### 3.5.1. Future Directions

Integration of exosome profiling into diagnostic and therapeutic strategies represents a shift towards precision women’s healthcare (Table 3). Regular assessment of exosomal biomarkers for early diagnosis and monitoring therapeutic response allows for real-time treatment adjustments and personalized medicine tailored to gynecological disorders.

#### 3.5.2. Standardization and Biomarker Validation

Clinical implementation requires standardization of exosome isolation, characterization, and biomarker validation protocols. Large-scale multicenter studies must establish population-specific reference ranges across diverse demographics, gestational ages, and clinical settings to validate exosomal biomarkers and demonstrate additional diagnostic value beyond existing methods. However, further work remains to reach a consensus for the standardization and validation of exosome utilization.

#### 3.5.3. Mechanistic Understanding and Multi-Omics Integration

A thorough understanding of tissue-specific exosome targeting, cargo sorting, and recipient cell responses will support rational therapeutic design. Combining multiple omics approaches—including proteomics, transcriptomics, lipidomics, and metabolomics—significantly improves diagnostic precision and facilitates personalized medicine strategies for pregnancy complications and gynecological disorders.

#### 3.5.4. Therapeutic Optimization and Manufacturing

To maximize the effectiveness of exosome use in disease diagnosis and treatment, systematic research on optimal exosome doses, administration routes, treatment schedules, and combination protocols is essential. Developing manufacturing methods that comply with good manufacturing practice and produce consistent, clinical-grade exosomes is crucial for therapeutic translation and regulatory approval.

#### 3.5.5. Regulatory Framework and Technology Integration

Regulatory agencies must establish clear pathways for exosome-based diagnostics and therapeutics with comprehensive safety monitoring systems. The integration of artificial intelligence and machine learning with multi-omics profiling, along with point-of-care detection devices, will accelerate the implementation of personalized reproductive healthcare.

## 4. Conclusions

Exosomes have transitioned from enigmatic cellular byproducts to key regulators of physiological and pathological processes in reproductive medicine. Their unique biogenesis through ESCRT-dependent and ESCRT-independent pathways produces vesicles with specific molecular signatures that mirror the physiological status of their originating cells, making them valuable diagnostic tools for disease detection. In obstetrics and gynecology, exosomes are involved in fundamental reproductive processes such as folliculogenesis, fertilization, embryo implantation, placentation, and labor, while disrupted exosomal communication underlies conditions ranging from infertility and pregnancy complications to gynecological malignancies. The ability to isolate and analyze exosomes from accessible biofluids like blood, urine, and follicular fluid offers unprecedented opportunities for non-invasive disease monitoring and early diagnosis. Exosomal biomarkers have shown promise in predicting preeclampsia, diagnosing endometriosis, evaluating gynecological cancer risk, and assessing embryo viability, providing significant benefits over traditional diagnostic methods. Beyond their diagnostic potential, mesenchymal stem cell-derived exosomes are emerging as cell-free therapies with regenerative properties for conditions such as premature ovarian insufficiency, Asherman syndrome, and other reproductive disorders, avoiding safety concerns associated with cellular therapies while maintaining biological effectiveness. However, translating exosome research into clinical practice faces major challenges, including standardizing isolation and characterization procedures, establishing reference ranges for biomarkers, understanding tissue-specific targeting mechanisms, optimizing therapeutic doses, and navigating regulatory processes for clinical use.

Future directions should emphasize large-scale multicenter validation studies to establish strong biomarker panels, develop engineering techniques to improve exosome targeting and cargo loading for therapeutic uses, incorporate multi-omics platforms for detailed exosomal content analysis, and explore exosome-based combination therapies for complex reproductive disorders. As analytical techniques advance and mechanistic insights deepen, exosomes are set to transform personalized medicine in obstetrics and gynecology, providing customized diagnostic and treatment options that target the molecular basis of individual diseases. The integration of exosome biology with artificial intelligence, innovative technology, and precision medicine platforms will likely speed up clinical translation, ultimately enhancing reproductive outcomes and gynecological health across diverse patient groups.

## Figures and Tables

**Table 1:** Exosome-Associated Biomarkers and Clinical Utility in Key OBGYN Conditions (Diagnostic focus).

Condition	Sample Source	Key Biomarkers/Cargo	Cargo Type	Clinical Utility	References
Infertility	Follicular fluid Serum	ENO1, HSP90B1, Fetuin-B, Complement C7, CD9, APOC4	Proteins	Diagnosis of insufficient folliculogenesis Assessment of oocyte quality and ovarian aging	[47,112]
Recurrent Pregnancy Loss (RPL)	Plasma	miR-185-5p	miRNA	Prediction of early RPL Indicator of impaired angiogenesis at maternal–fetal interface	[79]
Preeclampsia (PE)	Maternal plasma, serum or urine	miR-675-5p, miR-3614-5p, miR-520a-5p	miRNA, Proteins	Early diagnosis Prediction of disease severity (early vs. late onset) Monitoring placental dysfunction	[59,121,124,125,126]
Gestational Diabetes (GDM)	Maternal plasma or urineUmbilical cord blood	miR-99 family, circRNAs, Placental proteins	miRNA, circRNA, Proteins	Early prediction Differentiation from normal pregnancy Monitoring fetal metabolic impact	[32,135,136]
Polycystic Ovary Syndrome (PCOS)	Follicular fluid Serum	miR-379-5p, miR-143-3p, miR-155-5p, miR-323-3p, S100-A9	miRNA, Proteins	Diagnosis of granulosa cell dysfunction Monitoring ovarian inflammation Fertility assessment	[87,94,139,140,141,142]
Premature Ovarian Insufficiency (POI)	Serum Ovarian tissue	miR-205-5p (MSC-derived)	miRNA	Assessment of ovarian function restoration Monitoring angiogenic potential in therapy	[147]
Endometriosis	Peritoneal fluid Serum	lncRNA CHL1-AS1, miR-134-5p, miR-197-5p, miR-22-3p, miR-610	lncRNA, miRNA	Non-invasive diagnosis Monitoring disease progression and recurrence Distinguishing from other masses	[18,149,150,151]
Asherman Syndrome	Uterine tissue	MSC-derived anti-inflammatory factors	miRNA, Proteins	Monitoring endometrial regeneration and reduction in uterine fibrosis	[99,146]
Ovarian Cancer	Serum Ascites	circFoxp1, miR-221-3p, miR-1290, miR-99a-5p, CD44, CD47	circRNA, miRNA, Proteins	Early detection; prognosis prediction Identification of chemoresistance (e.g., platinum)	[19,60,67,107,153,154,155,156,157,158,159,160,161,162,163,164]
Endometrial Cancer	Uterine tissue Serum	lncRNA NEAT1, Tissue Factor (TF), CD144+ microparticles	lncRNA, Microparticles	Diagnosis Correlation with tumor stage and histological grade	[171,172,173]
Cervical Cancer	Cervix tissue Body fluid	miR-1286, lncRNA DLX6-AS1, miR-22	miRNA, lncRNA	Early diagnosis; prognosis assessment Monitoring lymph node metastasis	[174,175,176,177,178,179]

**Table 2:** Exosome-Associated Biomarkers and Exosome-Based Interventions in Key OBGYN Conditions (Therapeutic focus).

Condition	Sample Source	Key Cargo/Pathway	Experimental Model	Reported Therapeutic Effects	References
POI	Human umbilical cord MSC-derived exosomes	Hippo pathway, SMAD3–AKT signaling	Chemotherapy- or toxin-induced POI animal models, granulosa cell culture	Restored follicle number Improved ovarian hormone profiles Enhanced granulosa cell proliferation and reduced apoptosis	[35,64,144,145]
	Hypoxia-preconditioned MSC-derived exosomes	miR-205-5p to PTEN–PI3K–AKT–mTOR	Animal POI models	Enhanced angiogenesis and ovarian tissue repair Improved ovarian function parameters	[57,147,148]
	Human amniotic fluid MSC-derived exosomes	miR-369-3p/YAF2/PDCD5/p53 axis	Granulosa cell apoptosis models	Inhibited granulosa cell apoptosis Protected ovarian reserve in injury models	[82]
PCOS	MSC-derived exosomes (e.g., bone marrow/umbilical cord)	miR-323-3p and other regulatory miRNAs	PCOS animal models and in vitro granulosa cells	Improved granulosa cell proliferation Reduced apoptosis Partial normalization of ovarian morphology and hormonal milieu	[94,139,140,141,142]
Asherman syndrome	MSC-derived exosomes (e.g., menstrual blood, UC-MSC)	Anti-fibrotic factors; SMAD3/AKT/MDM2/p53 modulation	Rodent intrauterine adhesion models	Reduced endometrial fibrosis Increased endometrial thickness and vascularization Improved fertility outcomes	[99,146]
Endometriosis	Human umbilical cord MSC-derived exosomes	Pro-regenerative factors affecting epithelial migration	In vitro endometrial glandular epithelial cell models	Enhanced migration and repair capacity of endometrial cells Potential to modulate ectopic/endometrial balance	[35,64,149,152]
Ovarian cancer	Drug-incubated human MSC-derived exosomes	Antitumor drug cargo (e.g., triptolide or cytotoxics)	Ovarian cancer cell lines and xenograft models	Increased tumor targeting Reduced systemic toxicity Inhibition of tumor growth	[42,170]
	Tumor-derived or MSC exosomes	miR-221-3p, integrins, YBX1, gelsolin, etc.	In vitro and in vivo EOC/SOC models	Modulation of macrophage polarization, angiogenesis, and chemoresistance Potential therapeutic targets rather than direct therapies	[88,103,153,174]

**Table 3:** Exosome-Based Clinical Strategies in OB/GYN.

Clinical Implication	Exosome Source/Feature	Example
Screening	Serum, urine, follicular fluid	Early detection of PE, GDM, ovarian cancer, cervical cancer
Diagnosis	Specific exosomal miRNAs/proteins	Diagnosing PCOS, POI, endometriosis, cancer type/type staging
Prognosis/Monitoring	Circulating exosomal signatures	Predicting PE/GDM onset, cancer progression, recurrence
Therapeutic (Experimental)	Engineered exosomes (MSC, hUCMSC, loaded drugs)	Ovarian rejuvenation (POI), PCOS recovery, targeted drug delivery for cancers

## Data Availability

No new data were created or analyzed in this study. Data sharing is not applicable to this article.
